# Statement of Retraction: lncNALT knockdown ameliorates hypertensive retinopathy via PTEN/PI3K/AKT pathway

**DOI:** 10.1080/21655979.2026.2641952

**Published:** 2026-03-12

We, the Editors and Publisher of the journal *Bioengineered*, have retracted the following article from publication.

Kang, H., Huang, D., Li, H., Deng, X., Liu, S., Gou, W., … Yang, X. (2022). lncNALT knockdown ameliorates hypertensive retinopathy via PTEN/PI3K/AKT pathway. *Bioengineered*, 13(7–12), 15003–15012. https://doi.org/10.1080/21655979.2023.2180591

Since publication, significant concerns have been raised by a third party about the integrity of the data and reported results in the article. This includes image duplication with the following article:

Zhang, L., Wang, X., Che, W., Yi, Y., Zhou, S., & Feng, Y. (2022). Methyltransferase-like 3 silenced inhibited the ferroptosis development via regulating the glutathione peroxidase 4 levels in the intracerebral hemorrhage progression. *Bioengineered*, 13(6), 14215–14226. https://doi.org/10.1080/21655979.2022.2084494

When approached for an explanation, the authors provided their original data and answered our queries, but these do not sufficiently address the concerns.

As verifying the validity of published work is core to the integrity of the scholarly record, we are therefore retracting the article. The authors have been informed of this decision.

We have been informed in our decision-making by our policy on publishing ethics and integrity and COPE guidelines. 

The retracted article will remain online to maintain the scholarly record and will be digitally watermarked on each page as ‘Retracted’. 

